# Validation of a Deep Learning U-Net Algorithm for Multistructure Segmentation of Infrarenal Abdominal Aortic Aneurysms including Lumen, Thrombus, and Calcifications

**DOI:** 10.1016/j.ejvsvf.2025.11.001

**Published:** 2025-11-13

**Authors:** Lucie Derycke, Asmaa Doudouh, Florian Cotte, Abdelmalek Habi, Jean-Noel Albertini, Antoine Millon

**Affiliations:** a Department of Vascular Surgery, Hôpital Paris Saint-Joseph, Paris, France; b PrediSurge, St Etienne, France; c Vascular and Endovascular Surgery Department, Saint-Joseph Hospital, Marseille, France; d Department of Vascular and Endovascular Surgery, Hospices Civils de Lyon, Louis Pradel University, Bron, France

**Keywords:** Abdominal aortic aneurysm, Artificial intelligence, Automatic segmentation, Deep learning, Digital twin, Endovascular aortic repair

## Abstract

**Objective:**

Artificial intelligence and digital twin technologies provide a new way to plan endovascular interventions and can help practitioners anticipate complications. The accuracy of these methods is based on reliable automated aortic segmentation, including intraluminal thrombus (ILT), calcifications, and detection of collateral arteries. The aim of this study was to validate a new fully automated deep learning based aortic segmentation algorithm that could be used for optimised digital twin generation.

**Methods:**

After training on 1280 computed tomography angiography scans, including 1000 for pre-training and 280 for fine tuning, a convolutional neural network based on a U-Net architecture was externally validated on 48 computed tomography angiography scans to segment lumen, collateral arteries, ILT, and parietal calcifications of the abdominal aorta and iliac arteries. Blinded, manually corrected segmentations from a senior radiologist and surgeon were performed to create the ground truth comparison.

**Results:**

The comparison between fully automated and manually corrected segmentation methods revealed a mean dice similarity coefficient of 0.97 ± 0.01, 0.94 ± 0.05, and 0.87 ± 0.04 for aortic lumen, ILT, and calcifications, respectively. Average surface distance was 0.30 ± 0.15, 0.61 ± 0.72, and 0.28 ± 0.28 mm for aortic lumen, ILT, and calcifications, respectively. Mean segmentation time was four minutes and 20 seconds with the fully automated method.

**Conclusion:**

The deep learning algorithm developed in this study provided valid, fast, and accurate aorto-iliac segmentation. This may be used to automatically generate reliable aortic digital twins for endovascular aortic repair planning.

## Highlights

• Convolutional neural network (CNN) became the medical image segmentation reference
• The CNN provided accurate segmentation of arterial lumen, thrombus and calcification
• Correct automatic detection of 86% of the main collateral arteries was achieved
• Fully automatic complex aortoiliac segmentation is feasible and accurate

## INTRODUCTION

Over past decades, endovascular repair of abdominal aortic aneurysm (EVAR) has become the first line treatment for abdominal aortic aneurysm (AAA). One crucial step for the technical and clinical success of EVAR is accurate planning of the intervention to choose the most suitable endoprosthesis. This step is currently based on anatomic measurements on pre-operative computed tomography angiography (CTA) images. These measurements not only include aortic diameters, lengths, and angulations but also intraluminal thrombus (ILT) and parietal calcification at the levels of the aortic neck and iliac arteries.^1^^,^^2^

Modelling and segmentation of the aorto-iliac anatomy have received special interest due to the emergence of endovascular aortic therapy. Automated aortic segmentation techniques have emerged, leveraging machine learning and deep learning algorithms, to provide more accurate, consistent, and efficient results than those provided by manual and semi-automatic segmentation, which may be time consuming and subject to variability.^3^, ^4^, ^5^, ^6^, ^7^, ^8^ Such artificial intelligence (AI) algorithms have proved to be effective in segmenting the aorto-iliac volume and automatically detecting aortic lumen, ILT, and maximum aortic transverse diameter(s) accurately.^4^, ^5^, ^6^^,^^8^ These works underline the potential of AI to more accurately assess aneurysm sac evolution before and after EVAR.

Development of personalised models from patient data that estimate not only the arterial anatomy but also haemodynamics and biomechanics is capturing increasing attention. The emergence of AI and digital twins for enhanced planning of cardiovascular procedures has recently been highlighted.^9^ Particularly, digital twin technology has shown its potential in predicting aortic endoprosthesis behaviour by reproducing not only geometry but also material properties.^10^ Aorto-iliac deformations induced by the introduction of stiff guidewires and devices may also be anticipated.^11^^,^^12^ Significant advances in EVAR planning and outcome prediction have been demonstrated using a combination of digital twin and AI technologies.^13^, ^14^, ^15^, ^16^ ILT and parietal calcifications are present almost universally in AAAs and may significantly modify the mechanical behaviour of the aorto-iliac wall.^17^^,^^18^ Therefore, ILT and calcifications should be included in the aorto-iliac segmentation in order to generate even more realistic digital twins.^19^

Over the last decade, convolutional neural network (CNN) algorithms built upon U-Net architecture have emerged as highly adaptive segmentation models.^3^^,^^6^^,^^7^^,^^20^ This has now become the reference architecture for medical image segmentation due to its versatility across various tasks. Previous approaches to AAA segmentation have focused mainly on the lumen and thrombus, without addressing calcifications. To the authors’ knowledge, no algorithm has yet reported simultaneous segmentation of lumen, ILT, and calcifications from contrast enhanced CTA in a single model, probably due to the very different morphological patterns (extended lumen and thrombus *vs.* small punctate calcifications). A unified approach could reduce pipeline complexity, ensure spatial consistency, and provide clinically relevant data for digital twin applications in EVAR planning. This study presents a multiclass nnU-Net architecture approach that segments all three structures in a single step, including flow lumen, ILT, parietal calcifications, and major collateral arteries.

## METHODS

### Dataset

The study was conducted retrospectively from data obtained for multiple clinical purposes. It was performed in accordance with the Institutional Ethics Committee rules and followed the MR004 (*Méthodologie de Référence 004*) procedure from the national commission of data processing and freedoms regarding non-interventional studies. The study complied with the principles of the Declaration of Helsinki.

Validation data were collected retrospectively from different institutions. Both clinical sites used the same study protocol, which was reviewed and approved by the Institutional Review Board of Hospices Civils de Lyon (institutional review board number 00013204). Data processing steps were compliant with the European General Data Protection Regulation. Each participant was informed, and Digital Imaging and Communications in Medicine (DICOM) images were pseudonymised following the best current standards.

Inclusion criteria were patients presenting with an infrarenal AAA who had undergone pre-operative CTA with an arterial phase intravenous liquid contrast injection focusing on the aorto-iliac area.

Exclusion criteria were pararenal or thoraco-abdominal aneurysm, ruptured aneurysm, aortic dissection, non-contrast computed tomography (CT), presence of aorto-iliac stent or endoprosthesis, and poor quality imaging (>3 mm slice thickness) or the presence of artefacts that could compromise segmentation accuracy.

### Fully automated segmentation workflow

The training methodology was divided into two phases: an initial general training phase using an open source dataset, followed by a fine tuning phase using a specific set of images annotated by two experts.

#### Overview of the convolutional neural network U-Net algorithm

The algorithm employed a robust pre- and post-processing pipeline tailored to the dataset. During training, two primary loss functions, dice loss and cross entropy loss, were used to optimise performance. The Adam optimiser, with an adaptive learning rate, was used to accelerate the convergence of the model.^21^ Furthermore, *k* fold cross validation was implemented to ensure the model's robustness, with the internal dataset being divided into training and validation folds, whereas model performance was finally assessed on an independent external validation set. In this approach, the dataset is split into five folds, and the model is trained and evaluated five times, each fold serving once as the test set, whereas the remaining folds are used for training and validation. The segmentation framework was based on the nnU-Net v2.2 framework, a self-configuring 3D U-Net architecture for biomedical image segmentation that automatically adapts pre-processing, architecture, training, and post-processing to the task at hand.^22^ The model was configured to produce a three class output corresponding to the aortic flow lumen, ILT, and parietal calcifications.

Training and inference were performed using the nnU-Net implementation in PyTorch 2.7 (PyTorch Foundation, Linux Foundation, San Francisco, CA, USA) with CUDA 12.6 (NVIDIA Corporation, Santa Clara, CA, USA) on a workstation equipped with a NVIDIA GeForce RTX 4090 GPU (24 GB VRAM) (NVIDIA Corporation, Santa Clara, CA, USA) and an AMD Ryzen Threadripper PRO 5975WX CPU (Advanced Micro Devices, Santa Clara, CA, USA) under Ubuntu 22.04 (Canonical Ltd., London, UK).

#### Phase 1: Training on the TotalSegmentator dataset

The first training phase was performed on the TotalSegmentator dataset,^23^ which contains 1000 annotated CTA images focusing on the aorta and the iliac arteries (flow lumen only). The open source dataset covers a wide spectrum of vascular conditions, including healthy and diseased aortas, and incorporates scans from different institutions, scanners, and acquisition protocols.^24^ This dataset provided a broad foundation for the model to generalise on anatomic variations. The number of epochs was set at 1000, but this was adjusted based on the model convergence during training.

#### Phase 2: Fine tuning on expert annotated images

Once general training was complete, the model was fine tuned with a more specialised dataset consisting of 280 CTAs, including 167 cases from the same multicentre study as the test dataset and 113 cases selected from the open source dataset (restricted to scans containing the aorta). Overall, the dataset comprised 113 healthy aortas and 167 aneurysmal aortas, with 265 pre-operative and 15 post-operative scans. These images were manually annotated by one expert to include not only the flow lumen but also ILT and calcifications. The region of interest for segmentation extended from 10 cm above the coeliac artery to 3 cm below the iliac bifurcation. Collateral arteries (coeliac, superior mesenteric, and right and left renal arteries; polar renal artery[ies]; and right and left internal iliac arteries) were segmented up to a distance of 2 cm from the ostia.

For this phase, the training process converged after 300 epochs, as determined by an early stopping mechanism. The stopping criterion relied on the convergence of the validation loss, defined as a reduction of less than 0.02 over 50 consecutive epochs. This fine tuning step was crucial for refining the model's ability to segment complex structures such as ILT and calcifications, which were more challenging than the flow lumen.

#### External validation phase

The performance of the segmentation model was externally validated using five key metrics on a test set consisting of 48 CTAs focusing only on pre-operative AAA CTAs. The test set was fully independent from the fine tuning subset. CTA images were collected from five distinct institutions: Hôpital Marie-Lannelongue (*n* = 20), Hôpital Européen Georges-Pompidou (*n* = 8), Groupe Hospitalier Bretagne Sud (*n* = 7), Hôpital Pontchaillou (*n* = 3), and Hospices Civils de Lyon (*n* = 1). An additional nine CTAs originated from the open access TotalSegmentator dataset, for which centre information was not available.

For each CTA, manual semi-automatic segmentation of the aortic lumen, thrombus, and calcification was independently performed using the 3D Slicer (v 5.9.0) (Slicer Community, Brigham and Women's Hospital and Harvard Medical School, Boston, MA, USA) contour drawing tool.^25^ Manual segmentation was conducted independently by two senior expert physicians: one radiologist and one surgeon (L.D.) from two different institutions.

##### Overlap based metrics

The overlap based metrics included dice similarity coefficient (DSC), Jaccard index, Hausdorff per slice distance (HD), average surface distance, and volume similarity (VS). Metrics were defined as follows:

dice coefficient. This metric measures the similarity between the automated segmentation and the manual ground truth, providing a general indication of accuracy.

Jaccard index. This metric measures the similarity between the automated and reference segmentations as the ratio of the overlap between the two segmentations to their union.

Hausdorff distance per slice. This measures the maximum distance (per slice HD) and surface (3D HD) between the automatically and manually segmented contours on a per slice basis, highlighting discrepancies in boundary precision.

Average surface distance. This metric measures the average of all distances between pixels on the predicted object segmentation border and its nearest neighbour on the reference segmentation border.

Volume similarity. This evaluates the difference in volume between the automated and manual segmentations, giving insights into over- or undersegmentation.

These metrics were applied to both the general training and fine tuning phases to assess how well the model generalised and adapted to the specific anatomical structures of interest. Overall, the methodology emphasised a systematic approach to optimising deep learning models for medical image segmentation, leveraging both large scale and expert annotated datasets.

### Statistical analysis

Descriptive data were displayed as means with standard deviations and medians with interquartile ranges (quartile 1, quartile 3). All statistical analyses were performed using Python software (Python Software Foundation, Wilmington, DE, USA).

## RESULTS

### Training phase

**Figure 1:**
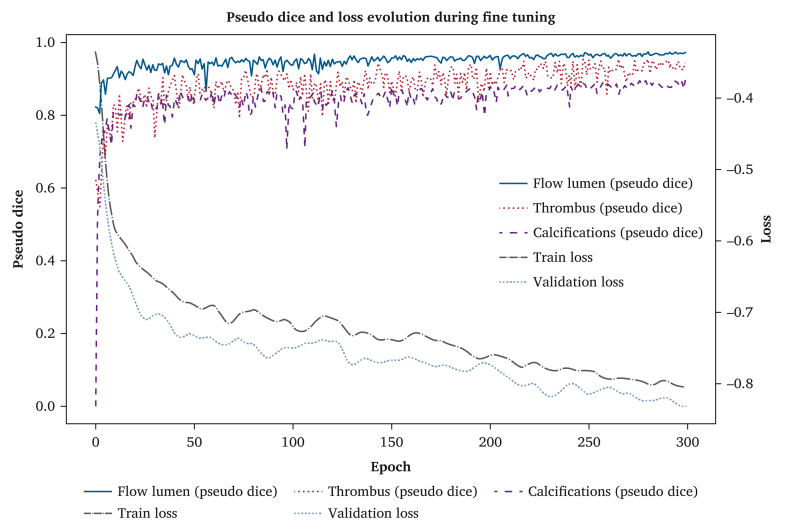
Evolution of pseudo dice metrics and loss during the fine tuning phase. *x*-axis: number of epochs (i.e., model iterations over the complete training dataset); *y*-axis: “loss” side: loss values measuring the error or divergence between model predictions and ground truth; “pseudo dice” side: pseudo dice metric indicating model performance in terms of segmentation quality. Metrics: training loss (dash dot line in purple), validation loss (dotted line with circle marker in orange), and pseudo dices for the flow lumen (solid line in red), the ILT (dotted line in brown), and calcifications (dashed line in blue).

The graph presented in Fig. 1 displays three main metrics: training loss, validation loss, and pseudo dices for the flow lumen, ILT, and calcifications. The training graph analysis showed favourable evolution of model performance. During the initial phase, a rapid decrease was observed in losses accompanied by an increase in pseudo dices, indicating effective learning across all structures. The slight fluctuations observed, particularly at the beginning of the training, were normal and diminished over time, confirming model stability.

Over subsequent epochs, both loss curves followed similar trajectories and gradually stabilised. The final validation loss converged to −0.8175 ± 0.0076, with a coefficient of variation of 0.92% over the last 50 epochs, confirming a highly stable optimisation process. This low variability (below the imposed criterion of 1%) further suggests that the model had reached a plateau without signs of overfitting.

The pseudo dices for the different anatomical components reached and maintained a high level of approximately 0.9, demonstrating excellent segmentation quality.

### External validation phase

A total of 18.362 slices were analysed by the algorithm and compared with the ground truth obtained from the manually corrected segmentations. The analysis encompassed aorto-iliac segmentation from 10 cm above the coeliac artery to 3 cm distal to the iliac bifurcation and up to 2 cm distal to the ostia of collateral arteries.

Mean volumes of arterial lumen, ILT, and parietal calcifications were 217.9 ± 64.3 cm^3^, 102.9 ± 74.2 cm^3^, and 13.1 ± 12.7 cm^3^, respectively. The mean time for automated segmentation was four minutes and 20 seconds per patient (range two minutes and ten seconds to 14 minutes and 40 seconds).

**Figure 2:**
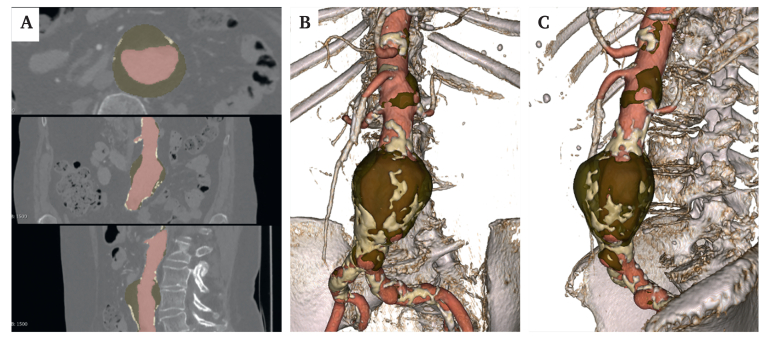
Representative images of the segmentation of the arterial lumen in red, intraluminal thrombus in brown, and parietal calcifications in light yellow. A Computed tomography angiography multiplanar sections from a patient with an infrarenal abdominal aortic aneurysm annotated with the fully automated segmentation. B, C Frontal and left sagittal views of the 3D aorto-iliac automated segmentation reconstructions.

Representative images of the fully automated segmentation of arterial lumen, ILT, main collateral arteries, and parietal calcifications are presented in Fig. 2.

**Figure 3:**
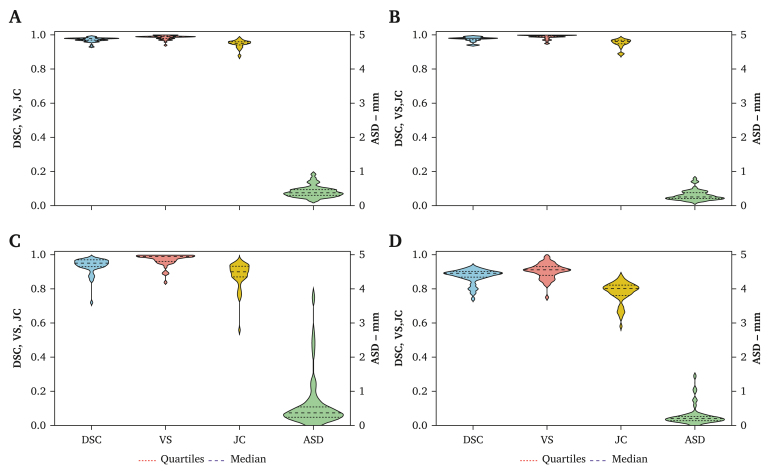
Summarised results of quantitative evaluation of automated segmentation compared with ground truth. A Global volume (flow lumen, intraluminal thrombus [ILT], and calcification volumes). B Flow lumen. C Intraluminal thrombus. D Calcifications. ASD = average absolute distance; DSC = dice similarity coefficient; JC = Jaccard Index; VS = volume similarity.

The results of the metrics used to evaluate the segmentation errors are presented in Table 1 and Fig. 3 for global volume (i.e., aortic lumen, ILT, and calcification volumes), aortic lumen, ILT, and calcification volumes.

**Table 1:** Summarised results of the evaluation of automated segmentation.

Variables	Aneurysmal aorta (*n* = 48)	
	Mean ± SD (min–max)	Median (IQR; Q1, Q3)
*Global*		
ASD, mm	0.42 ± 0.17 (0.15–0.99)	0.39 (0.31, 0.49)
DSC	0.97 ± 0.01 (0.93–0.99)	0.98 (0.97, 0.98)
JC	0.95 ± 0.02 (0.87–0.98)	0.95 (0.94, 0.96)
VS	0.99 ± 0.01 (0.94–1.00)	0.99 (0.98, 0.99)
HD per slice, mm	3.8 ± 9.1 (0–124.4)	1.5 (0.94, 2.3)
HD 3D, mm	26.5 ± 16.9 (10.1–96.6)	21.1 (15.7, 30.5)
*Flow lumen*		
ASD, mm	0.30 ± 0.15 (0.11–0.79)	0.26 (0.20, 0.38)
DSC	0.97 ± 0.01 (0.94–0.99)	0.98 (0.97, 0.98)
JC	0.95 ± 0.03 (0.88–0.98)	0.96 (0.95, 0.97)
VS	0.99 ± 0.01 (0.95–1.00)	0.99 (0.99, 1.00)
HD per slice, mm	3.5 ± 9.4 (0–124.4)	0.86 (0.78, 1.5)
HD 3D	24.9 ± 12.7 (8.8–70.3)	21.1 (15.8, 30.5)
*Intraluminal thrombus*		
ASD, mm	0.61 ± 0.72 (0.15–3.73)	0.36 (0.25, 0.53)
DSC	0.94 ± 0.05 (0.72–0.98)	0.95 (0.93, 0.97)
JC	0.88 ± 0.07 (0.56–0.97)	0.90(0.87, 0.968)
VS	0.97 ± 0.03 (0.84–1.00)	0.99 (0.96, 0.99)
HD per slice, mm	8.2 ± 10.0 (0–114.5)	4.2 (1.7, 11.8)
HD 3D, mm	41.7 ± 31.2 (9.3–172.8)	29.8 (22.1, 49.9)
*Parietal calcifications*		
ASD, mm	0.28 ± 0.28 (0.04–1.44)	0.20 (0.14, 0.26)
DSC	0.87 ± 0.04 (0.74–0.93)	0.89 (0.87, 0.90)
JC	0.78 ± 0.06 (0.58–0.87)	0.80 (0.76, 0.82)
VS	0.90 ± 0.04 (0.75–0.99)	0.91 (0.88, 0.93)
HD per slice, mm	7.9 ± 14.6 (0–121)	1.5 (0.81, 9.1)
HD 3D, mm	34.3 ± 34.5 (7.6–215.7)	22.8 (15.7, 39.1)

ASD = average surface distance; DSC = Dice similarity coefficient; HD = Hausdorff distance per slice; IQR = interquartile range; JC = Jaccard index; Q = quartile; SD = standard deviation; VS = volume similarity.

Overall, the single multiclass nnU-Net model produced a mean DSC of 0.97 ± 0.01, 0.97 ± 0.01, 0.94 ± 0.05, and 0.87 ± 0.04 for global volume, arterial lumen, ILT, and parietal calcifications, respectively. These results were obtained from a single inference pass of the model, which segments all structures simultaneously. For calcification segmentation, the model achieved an average specificity of 100% and an average sensitivity of 80%.

**Figure 4:**
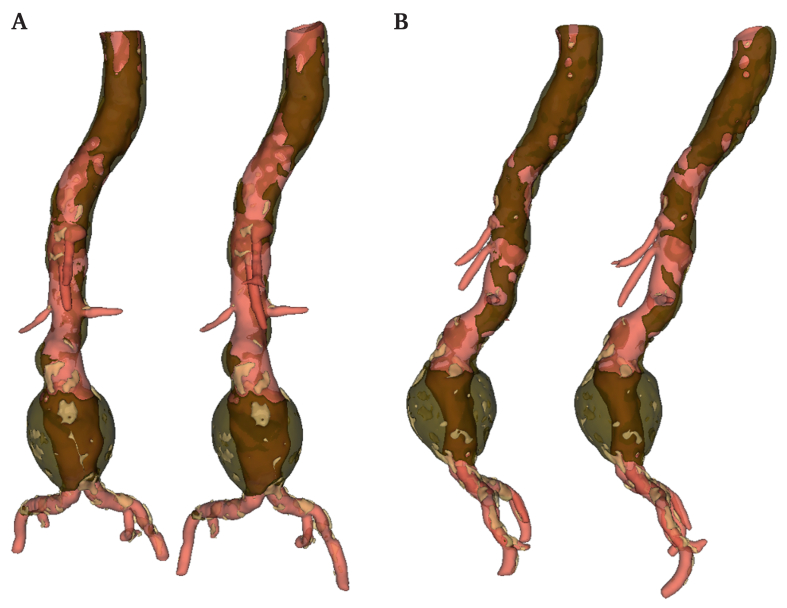
Representative images of 3D reconstructions of ground truth (left side) and fully automated segmentation (right side). A Front views. B Left sagittal views.

Representative images of the fully automatic and manually corrected segmentation are presented in Fig. 4.

To evaluate the contribution of pre-training, an ablation experiment was conducted by training the model from scratch (i.e., without using the TotalSegmentator dataset). The resulting dice scores were 0.97 ± 0.015 for flow lumen, 0.93 ± 0.07 for thrombus, and 0.86 ± 0.05 for calcifications.

A total of 246 of 286 (86.01%) main collateral arteries were correctly detected by the algorithm (i.e., the structure was segmented over a length greater than 1 cm and that the segmentation appeared visually correct when overlaid with the original CTA), including 100%, 97.9%, 74.8%, and 85.1% for the coeliac trunk, superior mesenteric artery, renal arteries (main and polar), and internal iliac artery, respectively.

The dice coefficients for lumen, ILT, and calcifications were 0.99, 0.99, and 0.99, respectively, between the two manual raters (interobserver agreement).

## DISCUSSION

This work demonstrated the precision and robustness of a fully automated segmentation algorithm using a validation set of 48 CTAs. The chosen architecture for this task was the nnU-Net framework, known for its adaptability to various medical image datasets. The key innovation of this work lies in the successful application of a single, multiclass nnU-Net model to segment three anatomically and radiologically distinct structures simultaneously: aortic flow lumen, ILT, and parietal calcifications in contrast enhanced AAA CTAs. To the best of the authors’ knowledge, this is the first study to report such performance in a one step, fully automatic workflow applied to AAA.

Although the TotalSegmentator dataset was used for pre-training purposes, the segmentation output from the TotalSegmentator algorithm itself was not suitable for direct use in the application. It does not distinguish between flow lumen, thrombus, and calcifications, and often lacks anatomic consistency, particularly between the aorta and iliac arteries, while omitting collateral branches. To assess the specific contribution of pre-training on TotalSegmentator, a model was trained from scratch without any pre-training phase. Although the results remained comparable, the model without pre-training showed slightly higher variability. These findings suggest that pre-training contributes modestly to segmentation robustness. However, as the volume and diversity of annotated training data continue to grow, the benefit of pre-training on external datasets is expected to become negligible.

Several AI technologies have recently emerged for automated segmentation of aneurysmal aortas, each offering unique strengths and limitations. Lareyre e*t al.*^8^^,^^26^ proposed a fully automated pipeline to detect aortic lumen, ILT, and calcifications with a boundary propagation and active contour method. The method was tested and validated on the same set of 40 patients and demonstrated good correlation for the aortic lumen and ILT. Nevertheless, no validation was reported regarding calcifications, and a limited number of slices were used to develop the model. By integrating advanced deep learning algorithms and fine tuned imaging techniques, a CNN based on U-Net architecture enables rapid, high fidelity segmentation of the aorta, addressing current limitations in speed, accuracy, and scalability. Lu *et al.*^27^ developed a 3D U-Net architecture model and reported good accuracy on a validation set of 57 CTs. Their algorithm was also used on non-contrast CT but was unable to detect the difference between lumen and ILT on contrast enhanced CT images. López-Linares *et al.*^28^ used a 3D CNN for AAA segmentation. Validation was achieved on 28 pre- and post-operative CTA images with promising results. One team reported results with a 3D CNN U-Net algorithm, with a first algorithm able to accurately segment thoraco-abdominal aortas with satisfying results on aortic lumen and ILT validation sets. However, the model depended entirely on ILT identification and segmentation.^29^^,^^30^ If ILT was not identified on the CTA images, no geometric analysis was performed. Then, the same team developed an algorithm to overcome this issue and demonstrated good accuracy to screen AAA, but no validation was reported with this new model.^7^ ILT regions, which are often small and thin, are not enhanced in CTA, making the boundary hard to delineate. To overcome this issue, Lyu *et al.*^31^ developed an alternative method using a multiscale U-Net based neural network named M^2^ Net workflow with promising results (DSC 0.88 in 30 testing cases). Lareyre *et al.*^32^ compared two methods, one using CNN with U-Net architecture, named expert system, and one hybrid method using machine learning trained with synthetic data obtained from the expert system. They noticed more accurate results with the hybrid approach (DSC 0.89 *vs.* 0.86 for ILT). Several companies have developed specialised solutions leveraging these scientific advances. Augmented radiology for vascular aneurysm (ARVA, Incepto Medical, Paris, France) focuses on deploying AI models that integrate seamlessly into clinical workflows, emphasising ease of use and reliability for entire thoraco-abdominal aorta diameter measurements.^5^^,^^6^^,^^33^ PRAEVAorta (Nurea, Bordeaux, France) specialises in a tool designed to analyse AAA diameter and volume changes before and after EVAR, with good results reported with contrast and non-contrast CTs.^3^^,^^4^^,^^34^^,^^35^ Finally, a recent study presented an advanced pipeline using a multistep nnU-Net approach for AAA segmentation for EVAR planning. The final model achieved a high segmentation accuracy for the aortic lumen and ILT (DSC 0.98 and 0.97, respectively) on a validation set of 20 CTAs. Agreement was lower for calcification (DSC 0.60).^36^

Previous studies have demonstrated the feasibility of CNN based segmentation of arterial calcifications, particularly on non-contrast CT and in non-aneurysmal settings. Most of the approaches focused on automated calcium scoring to derive a global quantification metric, without addressing a precise pixel level segmentation, necessary for advanced biomechanical modelling. Graffy *et al.*^37^ reported a fully automated vascular CNN calcification algorithm applied to non-contrast CT from patients undergoing colonography screening and demonstrated a good agreement value of 0.84 on a calcium score in the validation set against a semi-automated approach. Rau *et al.*^38^ proposed a model for thoracic and abdominal aorta analysis and achieved an *r* = 0.99 correlation for automated calcium quantification. On CTA images, distinguishing between contrast enhanced lumen and calcified plaques is more challenging. Advanced architectures with multiple networks are often needed (one for each label). Halkoaho *et al.*^39^ applied dual V-Net models on 58 CTAs and reported DSC and VS scores of 0.69 and 0.80, respectively, for automated detection of calcifications of non-aneurysmal aorto-iliac arteries. Weng *et al.*^40^ focused on superficial femoral artery calcifications on CTA with a 3D U-Net on 128 CTAs and achieved DSC 0.89. Bagheri Rajeoni *et al.*^41^ developed a pipeline for the entire arterial tree from the thoracic aorta to the popliteal arteries using ResNet-34 + U-Net, achieving DSC 0.83 for arterial segmentation and *r* = 0.98 for calcium score on 16 CTA. By contrast, the approach used in this study leverages a multiclass nnU-Net architecture capable of simultaneously segmenting the lumen, ILT, and parietal calcification. With a DSC of 0.87, Jaccard index of 0.78, and VS of 0.90, the results of this study demonstrate a good level of parietal calcification segmentation accuracy in patients with AAA on CTA images. Calcifications in direct contact with parietal thrombus may be easier to identify than those near a contrast enhanced lumen, due to the higher contrast gradient between calcium and thrombus.^26^ This anatomic configuration probably contributes to the good segmentation performance observed in the dataset. Nonetheless, the model was able to segment calcifications across a variety of spatial relationships with surrounding tissues and contrast filled lumen, highlighting its robustness and clinical applicability.

The results of the model are consistent with the literature on automated segmentation of the abdominal aorta, both in terms of computing time and accuracy. The mean time for automated segmentation was only four minutes per patient, compared with the reported mean time for manually corrected methods of 22–40 minutes.^3^^,^^4^^,^^26^

A single multiclass model was used throughout, rather than three separate CNNs, to enforce spatial consistency across class boundaries and reduce the risk of overlapping or inconsistent predictions. Despite the absence of calcifications in the pre-training dataset, fine tuning enabled the model to learn robust features for these structures from the internal data. This one pass, multiclass approach enhances both accuracy and robustness and simplifies the segmentation pipeline.

This distinction is particularly important for the development of digital twin technologies and automatic EVAR planification.^12^^,^^14^^,^^42^ Accurate segmentation of parietal calcifications is not only crucial for improving anatomical realism but also essential for better capturing the biomechanical heterogeneity of the aortic wall. Calcified plaques influence local stiffness and deformation patterns during endoprosthesis deployment and may significantly affect sealing and endoleak risks, especially type Ia and access related complications. By enabling precise integration of calcific burden into the simulation pipeline, the algorithm provides a foundational step toward the generation of personalised, predictive models that enhance procedure planning and device selection in EVAR. An automated objective assessment of iliofemoral artery calcification burden may also help to anticipate the risk of access related iliac and or femoral injuries. The accuracy of the model for these clinical applications remains to be validated. Future work will focus on evaluating the model's performance in automatically assessing ILT and parietal calcifications in the aortic neck and iliac arteries.

Visceral and internal iliac arteries were also segmented by the model with satisfactory accuracy. The feasibility of such segmentation was first explored by Fantazzini *et al.*,^29^ who applied a 3D CNN architecture to thoraco-abdominal CTAs in a small cohort of six patients. The limitation of this pipeline was the dependency on ILT identification to generate a geometric analysis. Riffaud *et al.*,^43^ using PRAEVAorta, presented a new method to automatically identify coeliac, superior mesenteric, renal, and common iliac arteries, with up to 89% of collateral arteries correctly detected and labelled. However, no precision was reported on the quality of the collateral artery segmentation. Lareyre *et al.*^44^ proposed a hybrid segmentation pipeline combining an expert based rule system with a supervised deep learning algorithm to extract the abdominal vascular tree and detect visceral artery aneurysms. In this study of 33 CTA scans, the system successfully identified 40 of 43 visceral artery aneurysms, demonstrating a high sensitivity (93%) and a moderate positive predictive value (51%). Recently, Robbi *et al.*^36^ reported the successful detection of all visceral branches and iliac arteries in 20 validation cases using the nnU-Net network and a flood filling algorithm. The approach used in this study relied solely on a fully automated CNN model without handcrafted features or secondary rule based filtering. It achieved correct identification of 86% of the main visceral branches using a multiclass nnU-Net segmentation framework. Automated detection of collateral arteries is mandatory to provide a solution relevant for automated planning. Moreover, inferior mesenteric artery (IMA) and lumbar artery patency is a well recognised risk factor for type II and delayed onset type I endoleaks following EVAR.^2^ Future developments will focus on improving detection of smaller or heavily calcified arteries, including inferior mesenteric and lumbar branches, which remain challenging for most current algorithms but are clinically relevant for accurate procedure planning.

This study was retrospective and focused on infrarenal AAAs. Only two experts and 48 pre-operative CTAs were considered as reference for ground truth comparison. Nevertheless, the level of validation and the results are consistent with previous literature on CNN based U-Net architecture for fully automated aortic segmentation. Moreover, the inclusion of CTAs originating from different institutions and different CT machines enables external validation on heterogeneous data, providing evidence of the algorithm's robustness.

The relatively lower dice score for calcifications may partly reflect annotation uncertainty rather than poor model performance. The analysis showed that false positives were rare and that most discrepancies were due to under segmentation (false negatives). The model often identified only the brightest areas of calcifications, whereas manual annotations sometimes included less dense peripheral regions. This selective behaviour aligns with the hypothesis that the model identifies well defined calcific cores while excluding lower density peripheral zones. Given the small size and irregular distribution of calcifications, defining an accurate ground truth remains inherently challenging.

The Hausdorff distance, per slice and 3D, aimed at highlighting discrepancies in boundary precision. Analysis showed some discrepancies, mostly due to the limits of segmentation that can be different between the automatic and the manually corrected segmentation, particularly at the level of the external iliac arteries. For example, if the automated segmentation stopped a few millimetres higher than the manual segmentation at the level of the iliac arteries, the reference for the Hausdorff distance calculation will be at the level of the contralateral iliac, resulting in discrepancies. This is amplified with the 3D HD, which captures the worst surface to surface distance in the entire volume. Consequently, the Hausdorff distance may reflect this positional mismatch rather than a true boundary error. Clinically, this should not be overinterpreted. The most critical anatomic region for EVAR planning remains the proximal aorto-iliac segment and aneurysm neck. Minor discrepancies in the distal external iliac contours may have little clinical impact on prosthesis sizing or clinical decision making.

## CONCLUSION

This study presents a fully automated deep learning algorithm for aortic segmentation, specifically targeting flow lumen, thrombus, parietal calcifications, and main collateral arteries. Future work will focus on developing improved segmentation of small collateral arteries and evaluating the model's performance in identifying relevant parameters for EVAR sizing, to provide a more comprehensive solution for planning and risk prediction.

## Funding

This work was supported by Agence Nationale de Recherche (ANR) RHU 5 EndoVx 881200212 and 10.13039/100018703European Innovation Council (EIC) Accelerator Project 190115232.

## CONFLICTS OF INTEREST

J.-N.A. is a cofounder of the company PrediSurge SAS. A.D. and F.C. are simulation engineers at PrediSurge SAS. A.H. is a data scientist at PrediSurge SAS. The other authors declare no competing interests.

## References

[1] Wanhainen A., Van Herzeele I., Bastos Goncalves F., Bellmunt Montoya S., Berard X., Boyle J.R. (2024). Editor's choice—European Society for Vascular Surgery (ESVS) 2024 clinical practice guidelines on the management of abdominal aorto-iliac artery aneurysms. Eur J Vasc Endovasc Surg.

[2] Chaikof E.L., Dalman R.L., Eskandari M.K., Jackson B.M., Lee W.A., Mansour M.A. (2018). The Society for Vascular Surgery practice guidelines on the care of patients with an abdominal aortic aneurysm. J Vasc Surg.

[3] Caradu C., Spampinato B., Vrancianu A.M., Bérard X., Ducasse E. (2021). Fully automatic volume segmentation of infrarenal abdominal aortic aneurysm computed tomography images with deep learning approaches versus physician controlled manual segmentation. J Vasc Surg.

[4] Caradu C., Pouncey A.-L., Lakhlifi E., Brunet C., Bérard X., Ducasse E. (2022). Fully automatic volume segmentation using deep learning approaches to assess aneurysmal sac evolution after infrarenal endovascular aortic repair. J Vasc Surg.

[5] Postiglione T.J., Guillo E., Heraud A., Rossillon A., Bartoli M., Herpe G. (2024). Multicentric clinical evaluation of a computed tomography-based fully automated deep neural network for aortic maximum diameter and volumetric measurements. J Vasc Surg.

[6] Adam C., Fabre D., Mougin J., Zins M., Azarine A., Ardon R. (2021). Pre-surgical and post-surgical aortic aneurysm maximum diameter measurement: full automation by artificial intelligence. Eur J Vasc Endovasc Surg.

[7] Spinella G., Fantazzini A., Finotello A., Vincenzi E., Boschetti G.A., Brutti F. (2023). Artificial intelligence application to screen abdominal aortic aneurysm using computed tomography angiography. J Digit Imaging.

[8] Lareyre F., Chaudhuri A., Flory V., Augène E., Adam C., Carrier M. (2022). Automatic measurement of maximal diameter of abdominal aortic aneurysm on computed tomography angiography using artificial intelligence. Ann Vasc Surg.

[9] Samant S., Bakhos J.J., Wu W., Zhao S., Kassab G.S., Khan B. (2023). Artificial intelligence, computational simulations, and extended reality in cardiovascular interventions. JACC Cardiovasc Interv.

[10] Derycke L., Avril S., Millon A. (2023). Patient-specific numerical simulations of endovascular procedures in complex aortic pathologies: review and clinical perspectives. J Clin Med.

[11] Gindre J., Bel-Brunon A., Rochette M., Lucas A., Kaladji A., Haigron P. (2017). Patient-specific finite-element simulation of the insertion of guidewire during an EVAR procedure: guidewire position prediction validation on 28 cases. IEEE Trans Biomed Eng.

[12] Daoudal A., Gindre J., Lalys F., Kafi M., Dupont C., Lucas A. (2019). Use of numerical simulation to predict iliac complications during placement of an aortic stent graft. Ann Vasc Surg.

[13] Derycke L., Avril S., Perrin D., Albertini J.-N., Cochennec F. (2022). Computer simulation model may prevent thoracic stent-graft collapse complication. Circ Cardiovasc Imaging.

[14] Derycke L., Avril S., Albertini J.-N., Vermunt J., Haulon S., Millon A. (2024). Patient specific numerical simulation of endovascular abdominal aortic aneurysm repair to predict type Ia endoleak. Eur J Vasc Endovasc Surg.

[15] Derycke L., Sénémaud J., Perrin D., Avril S., Desgranges P., Albertini J.-N. (2020). Patient specific computer modelling for automated sizing of fenestrated stent grafts. Eur J Vasc Endovasc Surg.

[16] Kliewer M.E., Bordet M., Chavent B., Reijnen M.M.P.J., Frisch N., Midy D. (2022). Assessment of fenestrated Anaconda stent graft design by numerical simulation: results of a European prospective multicenter study. J Vasc Surg.

[17] Demirci S., Manstad-Hulaas F., Navab N. (2009). Medical imaging 2009: visualization, image-guided procedures, and modeling.

[18] Witheford M., Borghese O., Mastracci T.M., Maurel B. (2022). An observational assessment of aortic deformation during infrarenal and complex endovascular aortic aneurysm repair. J Vasc Surg.

[19] Derycke L., Avril S., Vermunt J., Perrin D., El Batti S., Alsac J.M. (2024). Computational prediction of proximal sealing in endovascular abdominal aortic aneurysm repair with unfavorable necks. Comput Methods Programs Biomed.

[20] Ronneberger O., Fischer P., Brox T. (2015). U-Net: convolutional networks for biomedical image segmentation. Preprint.

[21] Kingma DP, Ba J. Adam: A Method for Stochastic Optimization, 30 janvier 2017, *arXiv*: arXiv:1412.6980. 10.48550/arXiv.1412.6980.

[22] Isensee F., Jaeger P.F., Kohl S.A.A., Petersen J., Maier-Hein K.H. (2021). nnU-Net: a self-configuring method for deep learning-based biomedical image segmentation. Nat Methods.

[23] Wasserthal J., Breit H.-C., Meyer M.T., Pradella M., Hinck D., Sauter A.W. (2023). TotalSegmentator: robust segmentation of 104 anatomic structures in CT images. Radiol Artif Intell.

[24] Wasserthal J. Dataset with segmentations of 117 important anatomical structures in 1228 CT images [dataset]. Available at: https://zenodo.org/records/10047292.

[25] Fedorov A., Beichel R., Kalpathy-Cramer J., Finet J., Fillion-Robin J.C., Pujol S. (2012). 3D slicer as an image computing platform for the quantitative imaging network. Magn Reson Imaging.

[26] Lareyre F., Adam C., Carrier M., Dommerc C., Mialhe C., Raffort J. (2019). A fully automated pipeline for mining abdominal aortic aneurysm using image segmentation. Sci Rep.

[27] Lu J.T., Brooks R., Hahn S., Chen J., Buch V., Kotecha G., Shen D., Liu T., Peters T.M., Staib L.H., Essert C., Zhou S. (2019). Medical image computing and computer assisted intervention—MICCAI 2019.

[28] López-Linares K., Stephens M., García I., Macía I., Ballester M.Á.G., Estepar R.S.J. (2019). Abdominal aortic aneurysm segmentation using convolutional neural networks trained with images generated with a synthetic shape model. Mach Learn Med Eng Cardiovasc Health Intravasc Imaging Comput Assist Stenting.

[29] Fantazzini A., Esposito M., Finotello A., Auricchio F., Pane B., Basso C. (2020). 3D automatic segmentation of aortic computed tomography angiography combining multi-view 2D convolutional neural networks. Cardiovasc Eng Technol.

[30] Brutti F., Fantazzini A., Finotello A., Müller L.O., Auricchio F., Pane B. (2022). Deep learning to automatically segment and analyze abdominal aortic aneurysm from computed tomography angiography. Cardiovasc Eng Technol.

[31] Lyu Z., Mu N., Rezaeitaleshmahalleh M., Zhang X., McBane R., Jiang J. (2024). Automatic segmentation of intraluminal thrombosis of abdominal aortic aneurysms from CT angiography using a mixed-scale-driven multiview perception network (M2Net) model. Comput Biol Med.

[32] Lareyre F., Adam C., Carrier M., Raffort J. (2021). Automated segmentation of the human abdominal vascular system using a hybrid approach combining expert system and supervised deep learning. J Clin Med.

[33] Wegner M., Fontaine V., Nana P., Dieffenbach B.V., Fabre D., Haulon S. (2025). Artificial intelligence-assisted sac diameter assessment for complex endovascular aortic repair. J Endovasc Ther.

[34] Coatsaliou Q., Lareyre F., Raffort J., Webster C., Bicknell C., Pouncey A. (2024). Use of artificial intelligence with deep learning approaches for the follow-up of infrarenal endovascular aortic repair. J Endovasc Ther.

[35] van Tongeren O.L.R.M., Vanmaele A., Rastogi V., Hoeks S.E., Verhagen H.J.M., de Bruin J.L. (2025). Volume measurements for surveillance after endovascular aneurysm repair using artificial intelligence. Eur J Vasc Endovasc Surg.

[36] Robbi E., Ravanelli D., Allievi S., Raunig I., Bonvini S., Passerini A. (2025). Automatic CTA analysis for blood vessels and aneurysm features extraction in EVAR planning. Sci Rep.

[37] Graffy P.M., Liu J., O'Connor S., Summers R.M., Pickhardt P.J. (2019). Automated segmentation and quantification of aortic calcification at abdominal CT: application of a deep learning-based algorithm to a longitudinal screening cohort. Abdom Radiol (Ny).

[38] Rau A., Michel L., Wilhelm B., Raghu V.K., Reisert M., Jung M. (2024). Deep learning to predict cardiovascular mortality from aortic disease in heavy smokers. Npj Cardiovasc Health.

[39] Halkoaho J., Niiranen O., Salli E., Kaseva T., Savolainen S., Kangasniemi M. (2024). Quantifying the calcification of abdominal aorta and major side branches with deep learning. Clin Radiol.

[40] Weng W., Ku Y., Chen Z., Zheng H., Xu C., Ding H. (2022). Superficial femoral artery calcification segmentation and detection in CT angiography using convolutional neural network. Comput Biol Med.

[41] Bagheri Rajeoni A., Pederson B., Clair D.G., Lessner S.M., Valafar H. (2023). Automated measurement of vascular calcification in femoral endarterectomy patients using deep learning. Diagnostics (Basel).

[42] Crawford S.A., Sanford R.M., Doyle M.G., Wheatcroft M., Amon C.H., Forbes T.L. (2018). Prediction of advanced endovascular stent graft rotation and its associated morbidity and mortality. J Vasc Surg.

[43] Riffaud S., Ravon G., Allard T., Bernard F., Iollo A., Caradu C. (2022). Automatic branch detection of the arterial system from abdominal aortic segmentation. Med Biol Eng Comput.

[44] Lareyre F., Caradu C., Chaudhuri A., Lê C.D., Di Lorenzo G., Adam C. (2023). Automatic detection of visceral arterial aneurysms on computed tomography angiography using artificial intelligence based segmentation of the vascular system. EJVES Vasc Forum.

